# Beginners' Mistakes in Tuberculosis Work

**Published:** 1913-08-16

**Authors:** 


### BEGINNERS' MISTAKES IN TUBERCULOSIS WORK.

BY A TUBERCULOSIS OFFICER.

The present season has certainly been a good one for phthisiologists; more have been fledged these last few months than used to be in as many years. Many trial flights are being made, and there have been one or two tumbles. Promoted school physicians, or gentlemen otherwise in municipal employ, have managed to put in their few weeks at a post-graduate course before being appointed to tuberculosis officerships and giving their views, in the correspondence columns of the *British Medical Journal*, as to the whole value of tuberculin, or some such simple subject. Medical men suddenly put in charge of new sanatoriums have found the task not, as expected, quite like driving a gig or commanding the Channel Fleet. That type of veteran with a well-earned high reputation—half consultant and half general practitioner—so common in the provinces has often been asked by responsible laymen to advise on points concerning perhaps the most intensively cultivated part of the entire field of internal medicine. A little didacticism (unobtrusive, let us hope) seems, therefore, not quite superfluous.

Not that there is the slightest lack of literature on consumption or tuberculosis generally. The trouble is, as the late Mr. Tom Smith observed of another department of medical work, that as a general thing those who write the books do not see —really see—the cases. For this and other reasons, such as the non-coincidence of sufficient real experience with sufficient literary power, the proper conducting, for instance, of a sanatorium has never been worthily described. If nothing else, want of space forbids the effort to do so here. But a better and much shorter way to put one's advice on this and kindred topics is to say not what should be done, but what should not.

### A Sanatorium "From the Life."

Let us draw from life the following picture. A fine, solid, impressive-looking building in large grounds and on expensive land, the whole costing a cool hundred thousand pounds. Its Harrison (remember "King Harrison," of Guy's) a distinctly capable and tactful, yet notoriously absolute, secretary; king by virtue of his great money-getting powers, and to all appearance very well paid himself. A large chapel, where came to preach every week-end a salaried chaplain, and where each new maid was duly confirmed. There were many of them, for the patients did no work, although they had entertainments and practised much irregular auto-inoculation through frequent singing. Troops of nurses, too, of every known grade, with at the head of the hierarchy a clerico-aristocratically minded matron enjoying a good deal of authority. Top-hatted visiting "honoraries," regarded as the sole repositories of technical knowledge, who thumped and listened weekly and prescribed much medicine. Lastly, a resident medical officer perforce remarkable for his self-adaptability. Now for a sample of the medical value of this medical institution.

One of the physicians had had under treatment at another sanatorium a patient whose high temperature refused to abate. So, quite rightly, for in Cornet's excellent if dogmatic work change of scene is recommended as a last-resort antipyretic, he transferred the case to the institution we are thinking of. Here it came under the notice of a nurse who happened to have been patient herself at a much smaller sanatorium run on Nordrach lines; and as the fever continued, in spite of openly expressed wishes to the contrary, she managed to get conveyed to the honorary, by some diplomatically circuitous route, her wish to treat the patient as Walther would have done. The great man's fancy was tickled at the idea of being led by a babe, and he consented. So the patient was kept strictly to bed instead of loafing about the ward. Every week there were head-shakings. To keep a consumptive in bed longer than three weeks was dangerous it was said; he might never get up again. Except for hæmoptysis, of course; then bed was imperative. Before long the temperature improved and one day reached normal. Well, nurse had fairly won; now of course the man could get up. But no, it was explained that the heterodox practice was to keep such people a further ten days recumbent and then begin with half an hour's sitting up. This was for a wonder allowed, and finally the case went out doing as well as any early one in the institution. So much for the medical side; and administratively this institution's career has latterly been very unsuccessful.

### The Real Lesson from Abroad.

Here, in fine, is a good negative from which to develop a photograph of sanatorium treatment. In the above all the whites should be blacks and the blacks whites. To begin with, the whole outset of affairs must be economical: the tuberculosis problem is first, last, and midst a financial one. To isolate advanced cases, to keep early ones in the sanatorium for the proper period, to feed their dependants meanwhile, to get after-care really started, will run away with every available penny. But a temporary building will cost more to keep up than a permanent one? So it will; but see how the labour of patients may be utilised: there are possibilities in this direction undreamed of even by Dr. Paterson. As to the technical part of the business, since the treatment came over here from Germany, introduced, not by our then current authorities on tuberculosis, but through a review article written by one Gibson, a cured patient, and as the second edition of Brehmer's book on the subject was published at Wiesbaden at a date (1889) when these gentlemen were preaching the deleteriousness of night air for consumptives, it would seem well to follow those who have modelled themselves on Continental lines from the first. Actual sanatorium practice is just the opposite of what even a medical man—one inexperienced in such places—would expect. Capillary hæmoptysis is comparatively little thought of if there be no pyrexia. Walther would even walk his patients for it. On the other hand, rise of temperature, even the slightest, receives instant attention; and as a result physiology has learnt that 98.4° Fahr. is no normal morning temperature. Medicine bottles are hardly ever seen.

Then as regards amusements, the treatment, especially now that graduated labour, tuberculin, and artificial pneumothorax have come in, absorbs pretty nearly all of everybody's energy, patients included. Latterly, too, the halfpenny morning newspaper is so widespread that even the poorest see it regularly. After all, as Mr. Gibson well said long ago, people come to a sanatorium to work at getting well, not to amuse themselves. Experienced heads know, too, that in reality the Abernethian manner is the kindest one. No convalescent, particularly a consumptive, can stand sympathy in continued doses without his character deteriorating and, indeed, his happiness suffering. Show him that you consider him a case for pity and he begins to pity himself and to complain. Occupy his mind with a bracing, vigorous "This do," and both he and the discipline of the institution benefit. Independent-minded people may not submit? But the true medical zealot is very swaying to sick persons; in their hearts patients like to be ordered. But if the exception crops up, then, as Emerson finely says, force must occupy until right arrives. The sense of it *will* arrive; the best, pleasantest, and most cheerful patients are often those who gave trouble at first, just as those who on admission profess great willingness to obey, or who seem pietistically inclined, are of doubtful omen. It is another of the multifarious paradoxes associated with this disease.

### Hints to the Officers.

Enough has perhaps been said to show that the work of treating consumption is not one any medical man can pick up quickly. And the obvious corollary is, What about your medical resident? Must he not be, as Dettweiler claimed years ago, rather exceptional? That is as it may be. But certainly, from the utilitarian point of view, he must not be stinted of professional or other authority. And as authority must be based upon something, must be deserved, the lesson for our newly fledged tuberculosis practitioners is to take care that nothing lacks to their competency. If they are to be really what they are styled, which is expert advisers to lay committees, they must have listened to a thousand consumptive chests and given five hundred tuberculin injections; should have done six months post-graduate work in laryngology—throat affections are awkwardly rife amongst advanced cases; must know something of artisans' wages and occupations; should have seen lung-collapse treatment; and they must read German. Quite a little will do for professional purposes—about as much (it may be admitted) as that amount of literary knowledge Byron describes as necessary in order to be able to quote.

Those initiating a sanatorium must remember too how much depends upon a right start. To the lay mind medicine is still quite undifferentiated—still in the Middle Ages. It thinks a fairly eminent medical man must needs know all there is to know concerning any branch of the subject. That pulmonary tuberculosis is already almost in the condition of an unrecognised specialty it is unaware; unaware, too, that our British hospital system of administration, rightly celebrated in its proper sphere, does not suit patients with an exclusively chronic and little disabling ailment. How few even of medical men know that Walther had no nurses! The bad effects of the adoption of wrong lines are not confined to the particular institution. Patients often enter more than one; various officials are transferred; and if incorrectly grounded to begin with, both will for a time feel and make it inconvenient in their new surroundings. More than this, of course, there is ahead of all recently instituted anti-tuberculosis work a period of severe political criticism. It behoves all concerned to keep this coming time of stress in mind, and meanwhile to make themselves as efficient as lies in their power.

